# Using electronic clinical decision support in primary care to enhance identification of primary aldosteronism: a protocol for the CONSEP cluster-randomised controlled trial

**DOI:** 10.1038/s41371-026-01207-9

**Published:** 2026-09-04

**Authors:** Grant Russell, Linghan Jia, Nigel Stocks, Mark R. Nelson, Jessie Edwards, Grace Jensen, Emiko Hunt, Stella May Gwini, Muhammad Akram, Heather Morris, Helen Skouteris, Gang Chen, Kenneth A. Sikaris, Michael Stowasser, Christopher M. Reid, John Malios, David Wyatt, Peter J. Fuller, Jun Yang

**Affiliations:** 1 Department of General Practice, School of Public Health and Preventive Medicine, Faculty of Medicine, Nursing and Health Sciences, Monash University, Melbourne, VIC Australia; 2 Centre for Endocrinology and Reproductive Health, Hudson Institute of Medical Research, Clayton, VIC Australia; 3 Discipline of General Practice, Adelaide Medical School, Adelaide University, Adelaide, SA Australia; 4 Menzies Institute for Medical Research, University of Tasmania, Hobart, TAS Australia; 5 Monash Centre for Occupational and Environmental Health, School of Public Health and Preventive Medicine, Monash University, Melbourne, VIC Australia; 6 Health and Social Care Unit, School of Public Health and Preventative Medicine, Monash University, Melbourne, VIC Australia; 7 Centre for Health Policy, Melbourne School of Population and Global Health, The University of Melbourne, Carlton, VIC Australia; 8 Healius Pathology, Sydney, NSW Australia; 9 Department of Pathology, University of Melbourne, Melbourne, VIC Australia; 10 Endocrine Hypertension Research Centre, University of Queensland Frazer Institute, Princess Alexandra Hospital, Brisbane, QLD Australia; 11 Curtin School of Population Health, Faculty of Health Sciences, Curtin University, Bentley, WA Australia; 12 Department of Epidemiology, School of Public Health and Preventive Medicine, Monash University, Clayton, VIC Australia; 13 Primary Aldosteronism Centre of Excellence Consumer Council, Hudson Institute of Medical Research, Clayton, VIC Australia; 14 Department of Molecular and Translational Science, Monash University, Clayton, VIC Australia; 15 Department of Medicine, Monash University, Clayton, VIC Australia

**Keywords:** Renovascular hypertension, Clinical trials, Diagnosis, Adrenal gland diseases

## Abstract

Primary aldosteronism (PA) is a common and treatable cause of hypertension, affecting up to 15% of patients in primary care, yet it remains substantially underdiagnosed. Recent international guidelines recommend routine screening for PA in all individuals with hypertension, highlighting the need for effective implementation strategies in general practice. We aim to determine whether an electronic clinical decision support (eCDS) tool integrated into general practice software improves PA screening and diagnosis. CONSEP (CONn Syndrome screening and Evaluation in Primary care) is a pragmatic cluster randomised controlled trial conducted in general practices across three Australian states. Practices will be block-randomised by size and location (state) in a 1:1 ratio to intervention (eCDS) or control. Patients eligible for analysis will be adults with hypertension attending participating practices. De-identified data will be extracted from clinical software at baseline, 12 months, and 24 months. Analyses will follow intention-to-treat principles using regression models accounting for clustering at the practice level. Primary outcomes are the proportion of patients screened for PA within 12 months and the proportion diagnosed with PA within 24 months. Secondary outcomes include blood pressure control, antihypertensive medication burden, cost-effectiveness, and a process evaluation assessing acceptability, feasibility, and sustainability. By embedding guideline-based screening into routine workflows, this trial addresses a major evidence-practice gap in hypertension care. If effective, the intervention could improve hypertension management, increase detection of PA, and reduce long-term cardiovascular risk in primary care.

## Introduction

### Background

Hypertension remains a leading cause of death and disability worldwide and is estimated to affect approximately 1.4 billion adults aged 30–79 years in 2024 [1, 2]. Despite its substantial health and economic burden, blood pressure (BP) control remains suboptimal internationally, ranging from 10% to 37% of those diagnosed with hypertension [3–6]. One major contributor to persistent poor BP control is failure to identify secondary causes of hypertension [7].

Primary aldosteronism (PA), also known as Conn Syndrome, is the commonest treatable cause of secondary hypertension [8]. It results from dysregulated aldosterone production from one or both adrenal glands, and is associated with a 2–4 fold higher risk of coronary artery disease, heart failure, atrial fibrillation, stroke and mortality than essential hypertension [9–11]. Timely diagnosis followed by targeted treatment, either adrenal surgery for unilateral disease or medical treatment with mineralocorticoid receptor antagonists, leads to improved BP control [12], regression of target organ damage [13, 14] reduction in cardiovascular risk [15, 16] and improved quality of life [17].

PA is increasingly recognised as a common condition in clinical practice, with prevalence estimates ranging from 5% to 14% among patients with hypertension in primary care settings [9, 18, 19]. Despite compelling evidence to screen people presenting with hypertension for PA, testing rates are far from optimal [20, 21]. European studies found only 3–7% of primary care hypertensive patients are screened for PA [22, 23], while in Canada, only 0.7% of hypertensive patients were screened [24]. With recent international hypertension and endocrine guidelines advocating for broader PA screening in all adults with hypertension [17, 25], interventions to improve PA screening in primary care, where most hypertension is managed, are urgently needed.

Screening for PA is most commonly performed using the aldosterone/renin ratio (ARR), a low-cost and widely available blood test with high diagnostic performance when appropriately applied [26, 27]. For individuals with a positive screening result, further evaluation with aldosterone suppression testing and targeted treatment is cost-effective and feasible in many settings, while empirical treatment with mineralocorticoid receptor antagonists may be appropriate in resource-limited environments [26, 28]. A key challenge is how best to support primary care clinicians to routinely order and interpret ARR tests.

Electronic clinical decision support (eCDS) systems have demonstrated benefit in improving recognition and management of hypertension in primary care [29]. Recent studies integrating eCDS into electronic health records have shown improvements in PA screening, with real-time prompts and decision support aiding test ordering and interpretation [30–33]. Screening rates post-intervention were variable, with a complex eCDS and prediction model tool achieving screening rates of 0.66%–2.2% [32], whereas simpler prompt-based systems increased screening by 15% above baseline in a large primary care population [31].

Systematic reviews indicate that multifaceted behaviour-change interventions, particularly those combining education, training and enablement, are effective at influencing general practice behavior [34]. Educational interventions and computerised reminders have also shown improved adherence to cardiovascular guidelines in primary care [35, 36]. However, it remains uncertain whether eCDS, when combined with routine clinician education, can meaningfully enhance PA screening and diagnosis in primary care.

The CONn syndrome Screening and Evaluation in Primary care (CONSEP) study has been designed to address this evidence gap. This protocol outlines a mixed-methods study that aims to evaluate whether implementation of an eCDS tool, in addition to standardised clinical education provided to both study arms, increases screening and diagnosis of PA among adults with hypertension attending Australian general practices. Concurrent implementation and cost-effectiveness evaluations will assess the feasibility, acceptability and value of adopting eCDS-supported PA screening at scale.

### Trial design

CONSEP is a pragmatic, parallel-group, cluster-randomised controlled trial.

## Methods and analysis

Reporting of the methods follows the Standard Protocol Items: Recommendations for Interventional Trials (SPIRIT) guidelines [37].

### Study setting

The study will be conducted in Australian general practices located in the states of Victoria, South Australia and Tasmania.

### Inclusion criteria

General practices will be included if they:

1. Provide comprehensive primary care services;
2. Intend to remain operational for at least two years following commencement of the intervention;
3. Have used a clinical management software system compatible with the eCDS tool for at least one year prior to enrolment and plan to continue using this software for the duration of the study;
4. Hold current accreditation with either the Royal Australian College of General Practitioners or the Australian College of Remote and Rural Medicine.

Practices that share a single patient database across multiple sites will be required to participate as a single cluster.

### Exclusion criteria

Practices will be excluded if they:

1. Participated in a prior PA educational intervention conducted by the study team [18];
2. Are previous or current users of the eCDS tool; or
3. Plan to implement the eCDS tool within the next two years.

### Study procedures

All participating practices, regardless of allocation, will receive a standardised PA and CONSEP education program delivered by CONSEP research officers. This will comprise a single 30-minute presentation covering clinical education on PA and indications for screening; an overview of the CONSEP trial; and an explanation of the standard diagnostic and referral pathway for PA.

**Fig. 1:**
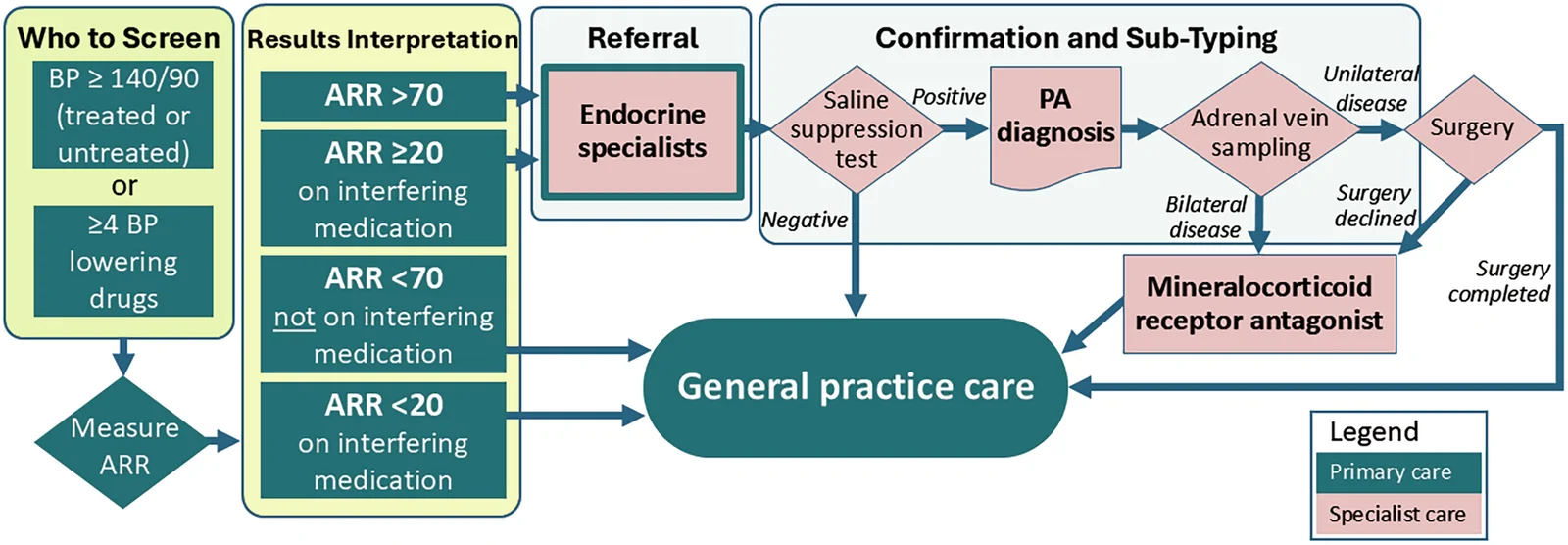
Diagnostic pathway following screening (primary care in teal, specialist care in pale pink; BP, blood pressure; ARR, aldosterone/renin ratio; interfering meds refer to antihypertensive drugs which affect the ARR. Interfering medication including: diuretics (e.g. hydrochlorothiazide, frusemide, indapamide), mineralocorticoid receptor antagonists (e.g. spironolactone, eplerenone), angiotensin-converting enzyme inhibitor (e.g. perindopril, ramipril), angiotensin II receptor blocker (e.g. irbesartan, telmisartan), dihydropyridine calcium channel blocker (e.g. amlodipine, lercanidipine).

General practitioners (GPs) in both arms will be advised to screen adult patients with newly diagnosed hypertension, and those with established hypertension and either BP ≥ 140/90 mmHg (with or without treatment) or on treatment with four or more antihypertensive agents. Participating GPs will be advised that patients with a positive ARR should be referred for further evaluation through public tertiary endocrine services or private specialist care. Test results will be returned to the referring GP and documented in the patient’s electronic health record. The diagnostic pathway following PA screening is shown in Fig. 1.

### Intervention

Practices randomised to the intervention arm will gain access to the eCDS intervention within one month of the PA education session. This includes installation of the eCDS software and user training.

The eCDS tool is a commercially available software integrated into the pathology ordering interface of general practice clinical management systems. It provides guideline-based test recommendations for a range of clinical conditions. For patients with hypertension or undergoing evaluation for hypertension, the tool recommends inclusion of the ARR within a panel of pathology tests. GPs will retain full discretion to select or remove recommended tests, or to use their preferred methods for ordering pathology tests, including using any pathology provider.

A supplementary handout detailing how to order ARR using the eCDS tool will be provided by research officers during the education session. Installation and a single user-education session will be delivered by the representative of the software provider on the same day, either in person or online, with ongoing technical support available as per the provider’s routine procedures.

### Outcomes

CONSEP’s primary outcomes will be measured at the level of the practice and will be calculated as being the difference between the intervention and control arms in: i) the proportion of eligible practice patients screened for PA within the first 12 months post-baseline; and ii) the proportion of eligible patients diagnosed with PA within 24 months post-baseline.

Secondary outcomes will include differences between the intervention and control arms in: i) BP control among eligible practice patients at 12 and 24 months; and ii) the number and dose of antihypertensive medications prescribed to eligible practice patients at 12 and 24 months.

Baseline will be defined as the date of the first education session in the control arm and the date of eCDS installation in the intervention arm.

Patients considered eligible for analysis will include adults who have attended the practice (in person or virtual) at least three times in the two years preceding the data extraction date *and* have at least two elevated BP recorded on separate dates (systolic BP ≥ 140 or diastolic BP ≥ 90), *or* a documented diagnosis of treated or untreated hypertension, *or* a current prescription for four or more antihypertensive medications. Patients with a prior diagnosis of PA or who have undergone testing for PA within the preceding 12 months will be excluded.

Screening for PA will be defined as the placement of a pathology request for aldosterone, renin and/or ARR, based on data extracted from the clinical software that collects such information.

Diagnosis of PA will be defined in two ways, clinical diagnosis and guideline-defined diagnosis. Clinical diagnosis will be based on a documented diagnosis or medical history of PA identified from the extracted data. For patients without a documented clinical diagnosis, a guideline-defined diagnosis of PA will be determined based on the 2025 Endocrine Society Clinical Practice Guidelines which states that PA is probable if plasma aldosterone ≥ 277 pmol/L, renin concentration ≤ 8.2 mU/L and ARR > 70 [17].

We will also collect implementation and economic outcomes, including acceptability, perceived usefulness, sustainability of the intervention among clinicians and practice staff; and cost effectiveness compared with the control arm.

### Participant timeline

**Fig. 2:**
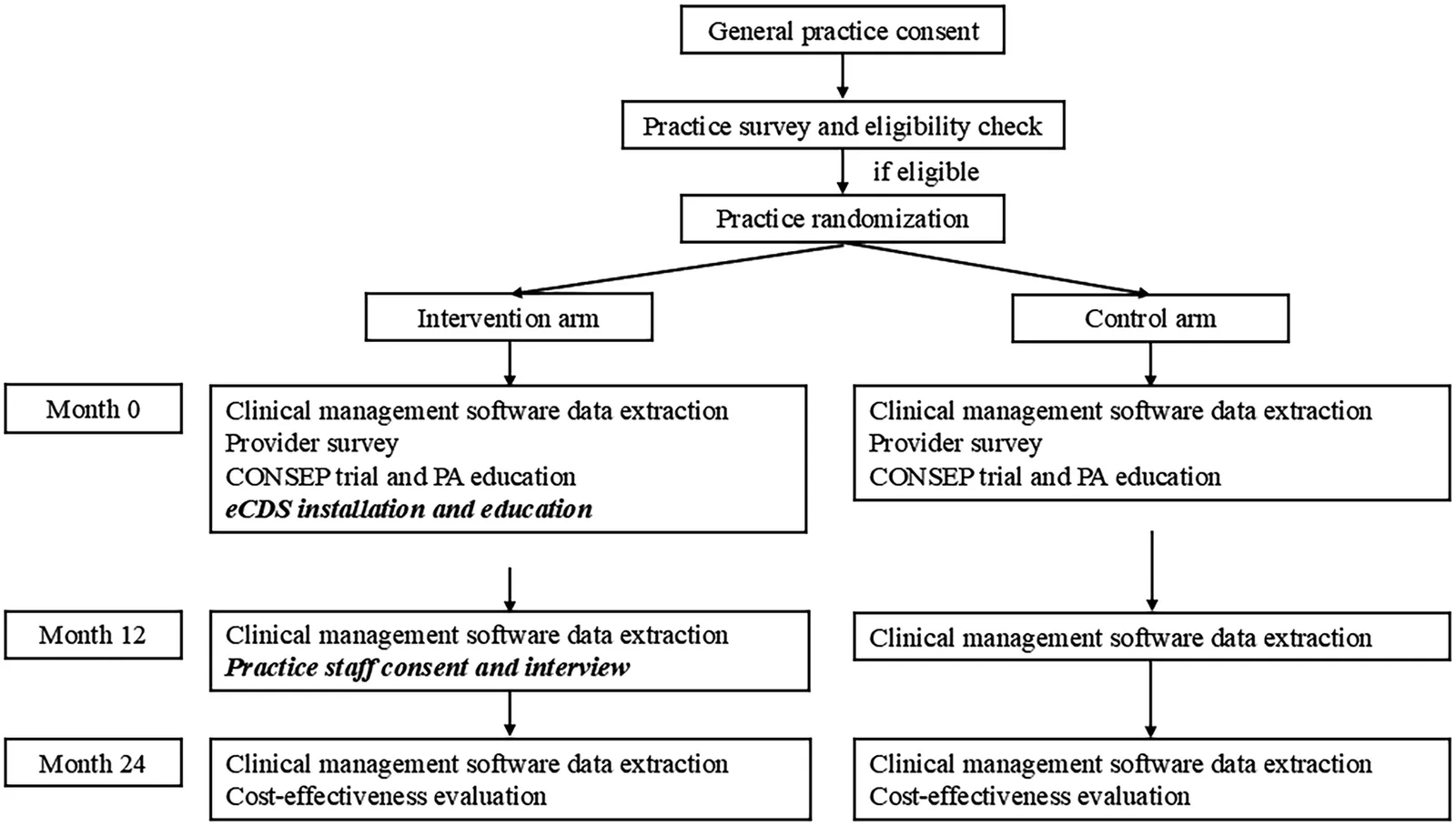
Schematic overview of the CONSEP trial timeline of the intervention arm and the control arm. The differences are shown in bold and italic text.

A timeline of the intervention arm and the control arm can be found in Fig. 2.

### Sample size

The study will recruit 28 clinics with 14 clinics randomised to each arm, providing an estimated 3,600 patients in each arm. Study power was calculated based on the two primary objectives, which are to compare screening and diagnosis rates between the two arms. Based on advice from primary care researchers and data from a prior PA screening study [18], it is estimated that 15% of eligible patients in the intervention arm and 2% in the control arm will be screened for PA in the 12 months post-intervention. In the same prospective screening study, 14% of screened patients were diagnosed with PA while < 0.1% of patients had a diagnosis of PA prior to active screening [18, 38]. We have allocated a more conservative rate of diagnosis in both study arms, estimating that ~7% and 0.3% of eligible patients in the intervention and control arm, respectively, are expected to be diagnosed with PA within 24 months (allowing 12 months from screening to diagnosis). The sample size will provide >90% study power for both screening and diagnosis outcomes. The study power is calculated presuming an intra-cluster correlation coefficient of 0.05, coefficient of variation in cluster sizes of 0.5, a two-sided test with 0.05 significance level, 20% dropout rate, and assuming 300 eligible patients per clinic over 12 months.

### Recruitment

Practices will be recruited through established research networks, including professional networks such as Practice Based Research Networks affiliated with University Departments of General Practice in each state, publicly available information, and institutional contacts, using mail, email, telephone, social media, and in-person invitations. Practices may also be identified through snowball sampling [39]. Initial contact will be made via the practice receptionist, practice manager or general practitioners. A $1500 reimbursement will be offered to the practices for their participation. Consent will be obtained at the general practice level to collect de-identified data.

### Allocation

Following recruitment, practices will be cluster-randomised to the intervention or control arm. Block randomisation with concealed allocation will be performed using a minimisation procedure. Minimisation factors will include practice size (grouped into two categories: number of full-time equivalent GPs working at the practice (< 5 vs. ≥ 5)) and practice location (Victoria, South Australia, Tasmania). The randomisation schedule will be prepared by an independent biostatistician not otherwise involved in the trial, using an allocation ratio of 1:1. Practices will be notified of allocation by the research officers.

### Blinding

Blinding of practices and study personnel will not be feasible due to the nature of the intervention, which includes training in the use of the eCDS tool. The trial statistician will remain blinded to treatment allocation until completion of the primary analysis.

### Data collection and management

Data collection will be conducted at the practice, provider, and patient (de-identified) levels. All data containing identifiable information relating to practices or clinicians will be de-identified by research officers prior to analysis. De-identified data will be stored on secure, institute-approved servers with access restricted to authorised research officers, the study manager, and the analysing statistician. Re-identification keys will be stored separately with restricted access.

Secure, institutionally approved systems will be used for data transfer and storage between participating practices and the research team.

### Baseline data collection for practice characteristics and eligibility

Practice-level data will be collected at baseline using the Demographics, Eligibility and Cost Assessment Measure (DECAM). This survey, with three sections, will be completed by research officers with assistance from practice managers or delegated staff. Sections A and B will be used to assess eligibility and describe practice characteristics such as their size and location. Section C will capture practice-level cost and billing information for the health economic evaluation. Data will be collected and stored in Research Electronic Data Capture (REDCap), a web-based electronic data collection system developed by Vanderbilt University and implemented via the REDCap Consortium [40, 41]. The local REDCap system used for this study was version 15.5.30, with access restricted to research officers and the study manager. Section C data will be de-identified prior to provision to the health economics team.

### Baseline data collection for primary care provider characteristics

General practitioners in participating practices will be invited to complete a Primary Care Provider Demographic Survey during PA and CONSEP education sessions. This survey will capture clinician demographics and prior education or experience related to PA screening and diagnosis. Survey data will be entered into REDCap by research officers and stored with restricted access.

### Clinical management software data extraction for baseline and follow-up data collection

De-identified patient-level data will be extracted from clinical management software using a purpose-built extraction script developed by the clinical software provider for this study. Extracted variables will include demographics (birth year, sex, gender, ethnicity), cardiovascular risk factors (smoking, alcohol use, family history, BMI, comorbidities), BP, pathology results, antihypertensive medications, and attendance data, consistent with predefined inclusion and exclusion criteria. Data extraction will be performed on site by the practice manager or delegate at baseline, 12 months post-baseline, and 24 months post-baseline.

Extracted datasets will be uploaded to a secure online drive with restricted access.

### Additional data collected for implementation and economic evaluation

#### Education attendance list

Attendance lists will be maintained for PA and CONSEP education sessions (recorded by research officers), and eCDS user training sessions in intervention practices (recorded by the practice manager or delegate). Attendance data will be used to assess fidelity and reach of the intervention and are stored securely with restricted access.

#### Education costs

Costs associated with PA, CONSEP, and eCDS education sessions will be documented by research officers. Education cost data will be derived from attendance records and session characteristics, de-identified, and provided to the health economics team for cost-effectiveness analysis.

#### Implementation change log

Research officers will maintain an implementation change log throughout the trial, documenting dates of education delivery, number of GPs trained relative to practice size, key questions or issues raised by clinicians, and contextual notes relevant to the implementation. These data will support interpretation of implementation outcomes and process evaluation.

#### Qualitative interviews

The process evaluation will be informed by semi-structured interviews conducted approximately 12 months post-baseline with participating GPs, practice managers, and staff involved in implementing the eCDS in intervention practices. Interviews will explore acceptability, appropriateness, feasibility, and perceptions of contextual factors influencing implementation, including team dynamics, workflows, and resource needs.

In addition, study research staff will be interviewed to explore recruitment processes, implementation challenges, communication, and perceived facilitators and barriers.

### Data monitoring

Given the minimal risk nature of the study, recommendation of established and safe investigations and referrals, absence of direct patient contact, and use of de-identified data only, a formal data monitoring committee will not be established.

### Harms

This study does not involve high-risk procedures, vulnerable populations, or direct interaction with patients. A potential harm of eCDS is automation bias, whereby clinicians may over-rely on software recommendations without adequately considering the individual patient context [42]. To mitigate this, GPs will receive upfront education emphasising that recommendations are advisory and must be applied using independent clinical judgement.

### Auditing

The study will be subject to auditing by the Monash University Human Research Ethics Committee.

### Statistical methods

Prior to data analysis, data imputation for missing values will be considered depending on the percentages of missing data. Patient level data will be analysed by intention to treat. Categorical data will be summarised using frequencies and percentages. Continuous data will be described using means (with standard deviation) if normally distributed or median (with lower and upper quartiles) if the distribution is skewed. The primary outcomes will be compared between study arms using Poisson regression with Huber-White sandwich estimators for errors. Results will be presented as risk ratios with 95% confidence intervals. The same regression model will be used for dichotomous secondary outcomes such as proportion with BP under control and resistant hypertension. Linear regression will be used for between-group comparisons of continuous outcomes such as dose of antihypertensive medications and BP. Quantile regression will be considered in cases where the assumptions of linear regression are violated. Additional analyses using multilevel modelling, to account for clinic and doctor level clustering, will be explored to ensure clustering is appropriately considered. Subgroup analyses will also be performed in participants with clinical features suggestive of primary aldosteronism, based on high-risk groups identified in the 2016 Endocrine Society Clinical Practice Guideline. These include individuals with blood pressure >150/100 mmHg, resistant hypertension, hypertension with hypokalaemia, an adrenal adenoma, obstructive sleep apnoea, or hypertension at a young age (<20 years) [26]. A *p*-value < 0.05 (two tailed) will be considered statistically significant. No missing data imputation will be employed. A detailed statistical analyses plan will be prepared by the lead statistician before data analyses is commenced. For all regression models, appropriate consideration of factors to adjust for are considered a priori and are listed in the statistical analyses plan.

### Implementation evaluation

All implementation evaluation data will be analysed using NVivo 15 (QSR International). Interviews will be audio-recorded, transcribed verbatim, and de-identified using unique codes. Audio files and transcripts will be stored in password-protected folders on secure servers with access restricted to the qualitative researcher. A hybrid deductive-inductive thematic analysis guided by the RE-AIM framework will be used to analyse the interviews, project documentation and field notes [43]. The deductive approach will be based on RE-AIM themes while inductive coding identifies data-driven codes. These codes will be iteratively revised and subsequently clustered into sub-themes and themes in the approach outlined by Nowell et al. [44]. Data triangulation methods (of quantitative and qualitative data) will compare the extent to which, and in what ways, results converge, diverge, and relate to each other to produce a more complete understanding of the data.

### Economic analysis

A within-trial health economic evaluation from a health care system perspective will be conducted using a cost-effectiveness analysis, with the primary and secondary outcomes used to measure effectiveness. For each participating clinic, costs associated with the education program will be collected using a standardised activity-based costing template by the research officers. Research costs will be excluded from the analysis. Healthcare cost, such as hypertension management, PA screening, and diagnosis costs will be estimated based on the utilisation data from trial data extracts. The incremental cost-effectiveness ratios of the intervention versus control arm will be calculated. We will further explore the long-term cost-effectiveness of improved PA screening in Australia by using a Markov model, building upon one that our team has previously published [32]. The trial outcome will supplement estimates from an updated comprehensive literature review on the relevant long-term effectiveness (measured using quality-adjusted life years) and healthcare costs associated with disease progression. Both costs and benefits will be discounted at 5% in the base model, in line with Medical Services Advisory Committee (MSAC) and Pharmaceutical Benefits Advisory Committee (PBAC) guidelines in Australia. Both one-way and a probabilistic sensitivity analyses results will be presented. A cost-effectiveness acceptability curve will be plotted, providing information about the probability that the intervention is cost-effective. The economic evaluation will be conducted using TreeAge Pro (TreeAge Software, Williamstown, MA; software available at http://www.treeage.com).

### Consent or assent

For practices, written informed consent will be collected from the practice leadership team or their delegate, while practice staff participating in in-depth interviews will be asked for written or oral consent.

### Ethical approval and trial registration

This trial protocol version 7.1, 26/Feb/2026 has been approved by Monash University Human Research Ethics Committee (ID: 39778), Adelaide University Human Research Ethics Committee (ID: 38069) and University of Tasmania Human Research Ethics Committee (ID: 29596) and has been registered with the Australian New Zealand Clinical Trials Registry (ACTRN12623001340628).

Any further change to the protocol that affects the trial design or scientific intent will be submitted for approval to the above Human Research Ethics Committees as an amendment.

## Discussion

Hypertension remains a major global public health challenge and a leading contributor to cardiovascular disease, stroke, and all-cause mortality [45, 46]. PA affects up to one in seven patients with hypertension in primary care, yet screening rates remain persistently low [47, 48]. The 2024 European Society of Cardiology (ESC) Hypertension Guidelines and the 2025 Endocrine Society Clinical Practice Guidelines for Primary Aldosteronism now suggest PA screening for all adults with confirmed hypertension, underscoring the urgent need for effective implementation strategies [8, 17, 25].

The CONSEP trial will be the first intervention study in the Australian primary care setting to evaluate whether an eCDS tool integrated within general practice software can improve PA detection and management. By embedding evidence-based recommendations directly into routine clinical workflows, this trial will address a critical evidence-to-practice gap in hypertension care.

Importantly, assessment of clinical effectiveness will be complemented by formal evaluation of implementation processes and cost-effectiveness, providing essential information to support scalability and policy translation. This integrated approach will inform how guideline-recommended PA screening can be operationalised in real-world primary care settings.

If proven effective, the intervention will have the potential to improve hypertension management, enable earlier diagnosis of a common and treatable condition, reduce cardiovascular and kidney complications, and lower long-term healthcare costs.

## Summary

### What is known about the topic:

- Primary aldosteronism is common but substantially under-diagnosed in primary care.
- Accurate early diagnosis of primary aldosteronism enables targeted treatment with adrenalectomy or mineralocorticoid receptor antagonists, improving BP control and long-term cardiovascular outcomes.

### What this study adds

- This cluster randomised controlled trial evaluates whether an eCDS tool integrated into general practice software will improve the detection and management of primary aldosteronism in Australian primary care.
- If effective, this intervention will provide an implementable strategy to improve hypertension management and reduce cardiovascular risk at scale.
